# Bioactive fractions and compound of *Ardisia crispa* roots exhibit anti-arthritic properties mediated via angiogenesis inhibition in vitro

**DOI:** 10.1186/s12906-021-03341-y

**Published:** 2021-06-25

**Authors:** Joan Anak Blin, Roslida Abdul Hamid, Huzwah Khaza’ai

**Affiliations:** Department of Biomedical Science, Faculty of Medicine and Health Sciences, Universiti Putra Malaysia, 43400 Serdang, Selangor Malaysia

**Keywords:** *Ardisia crispa*, Angiogenesis, Rheumatoid arthritis, Human umbilical vein endothelial cells, Human fibroblast-like synoviocytes for rheumatoid arthritis

## Abstract

**Background:**

*Ardisia crispa* (Thunb.) A.DC (Primulaceae), is a medicinal herb traditionally used by Asian people as remedies to cure inflammatory related diseases, including rheumatism. The plant roots possess various pharmacological activities including antipyretic, anti-inflammation and antitumor. Previous phytochemical studies of the plant roots have identified long chain alkyl-1,4-benzoquinones as major constituents, together with other phytochemicals. Hexane fraction of the plant roots (ACRH), was previously reported with anti-angiogenic and anti-arthritic properties, while its effect on their anti-arthritic *in vitro*, is yet unrevealed. Considering the significance of angiogenesis inhibition in developing new anti-arthritic agent, thus we investigated the anti-arthritic potential of *Ardisia crispa* roots by suppressing angiogenesis, in vitro.

**Methods:**

*Ardisia crispa* roots hexane extract (ACRH) was prepared from the plant roots using absolute *n*-hexane. ACRH was fractionated into quinone-rich fraction (QRF) and further isolated to yield benzoquinonoid compound (BQ), respectively. In vitro experiments using VEGF-induced human umbilical vein endothelial cells (HUVECs) and IL-1β-induced human fibroblast-like synoviocytes for rheumatoid arthritis (HFLS-RA) were performed to evaluate the effects of these samples on VEGF-induced HUVECs proliferation and tube formation, and towards IL-1β-induced HFLS-RA proliferation, invasion, and apoptosis, respectively. Therapeutic concentrations (0.05, 0.5, and 5 μg/mL) tested in this study were predetermined based on the IC_50_ values obtained from the MTT assay.

**Results:**

ACRH, QRF, and BQ exerted concentration-independent antiproliferative effects on VEGF-induced HUVECs and IL-1β-induced HFLS-RA, with IC_50_ values at 1.09 ± 0.18, 3.85 ± 0.26, and 1.34 ± 0.16 μg/mL in HUVECs; and 3.60 ± 1.38, 4.47 ± 0.34, and 1.09 ± 0.09 μg/mL in HFLS-RA, respectively. Anti-angiogenic properties of these samples were verified via significant inhibition on VEGF-induced HUVECs tube formation, in a concentration-independent manner. The invasiveness of IL-1β-induced HFLS-RA was also significantly inhibited in a concentration-independent manner by all samples. ACRH and BQ, but not QRF, significantly enhanced the apoptosis of IL-1β-induced HFLS-RA elicited at their highest concentration (5 μg/mL) (*P* < 0.05).

**Conclusions:**

These findings highlight the bioactive fractions and compound from *Ardisia crispa* roots as potential anti-arthritic agents by inhibiting both HUVECs and HFLS-RA’s cellular functions in vitro, possibly mediated via their anti-angiogenic effects.

**Supplementary Information:**

The online version contains supplementary material available at 10.1186/s12906-021-03341-y.

## Background

Rheumatoid arthritis (RA) is an inflammatory/autoimmune joint degenerative disease causing permanent deformities if not actively treated [1]. While the disease etiology remains unknown, therapeutic interventions mainly focused on controlling inflammation, reducing pain, preserving the joint function and improving the patients’ quality of life (QoL) [2]. While non-steroidal anti-inflammatory drugs (NSAIDs) were an important tool in the treatment of RA, they are currently used as “bridge” until the disease-modifying antirheumatic drugs (DMARDs) start to produce therapeutic effects [3, 4]. Of note, biologic DMARDs have revolutionized the treatment of arthritis to treat-to-target approach. Yet, these treatment options are challenging as their application need proper consideration between their efficacies and the treatment-specific side effects [5].

Angiogenesis, a process in which new vessels sprouting from preexisting vasculature, is a potential target for therapeutic interventions in RA [6]. It is commonly a physiological process at its equivocal balance, such as organ development, tissue repair, and reproduction. However, when there is an imbalance between its pro-angiogenic and its anti-angiogenic factors, it will lead to significant pathological consequences, resulting in either excessive or insufficient angiogenesis [7]. Excessive angiogenesis, due to excessive new blood vessel outgrowth will lead to the progression of several human diseases, as implicated in the early pathogenesis of RA [8]. During RA courses, excessive angiogenesis is the critical initial event supporting pannus growth of the rheumatoid synovium, which ends with the destructive joints [9]. As strong correlations exist between angiogenesis and RA; therefore, many anti-arthritic drugs currently in the phase of development are designed to target angiogenesis inhibition for RA therapeutics [10].

Biologic therapies are types of drugs that directed at specific target of immune response components [10, 11]. To date, the emergence of biologic RA therapies targeting on pro-angiogenic signaling pathways, has greatly improved for treatment in most RA patients. TNF-α inhibitors (infliximab), anti-IL-1 receptors (Anakinra), and anti-IL-6 receptors (tocilizumab), are amongst available biologic agents used to target specific mediators in RA angiogenesis [11, 12]. Although these biologics application in clinical practice has grown constantly, however, their use is highly variable across the nation, where the costs remain the major barrier [13]. Due to this limitation, considerable efforts have been dedicated for developing less expensive anti-arthritic agents as an alternative to these biologics, particularly from plant sources [14–16].

Since prehistoric times, humans have been using plants as their medicine to alleviate and treat diseases [17]. Plant derivatives are small molecules that could be directed as potential health-promoting agents in modern medicine. It is postulated that the small molecules which could interfere with the pro-angiogenic signaling pathways mimicking the biologics are possible for RA therapeutics. Quinones are amongst the earliest types of small molecules that have been premeditated as hypoxia-selective tumor-activated prodrugs [18]. These plants’ secondary metabolites consist of benzene rings with one or more hydroxyl substituents [19]. Having this structure makes quinones powerful as health-promoting agents [20].

*Ardisia* species, distributed throughout tropical and subtropical regions of the world is medicinal plants rich in quinones, together with other major phytoconstituents include polyphenols, triterpenoid saponins, isocoumarins and alkylphenols [21]. *Ardisia crispa* (Thunb.) A. DC. (Primulaceae), locally known as ‘*Mata Itik*’ or ‘hen’s eyes in Malaysia, is one of the biologically active specie found in the Asian region [21, 22]. Both leaves and root parts of this plant have been implemented in traditional practices by the local villagers. The root is used as decoction in treating fever, swelling, pain, rheumatism, and blood circulation [23]. Whilst, its leaves are crushed and applied at the affected sites as an antidote for scorpion and snake bites [24]. The root is mixed with other plants to treat women with dysmenorrhea in Thailand [25]. In Indo-China region, the locals treat chest illnesses with the plant root extract while Taiwanese use it as diuretic and antidote for poison [23]. These traditional uses have grabbed attention for its therapeutic potential in modern medicine.

Previous phytochemical studies of *Ardisia crispa* have characterized two utero-contracting triterpenoid saponins (ardisiacrispin A and ardisiacrispin B) from the plant’s root [24]. The root part has also been reported to contain a benzoquinonoid compound, precisely 2-methoxy-6-tridecyl-1,4-benzoquinone, with the documented antimetastatic and antitumor effects [26]. In a different study, Huang [27] elucidated 11 other compounds of *Ardisia crispa* roots, i.e., wogonin, oroxylin A, wogonoside, baicalin, (+) anwulignan, *meso*-dihydroguaiaretic acid, 4-hydroxyvaleric acid, *n*-tetradecane, bergenin, β-sitosterol, and ardisiacrispin C. The compounds (wogonoside and ardisiacrispin C) were reported to possess antitumor promoting activity [27]. *Ardisia crispa* roots have also been identified with an isomeric mixture of viminalol (also known as α- and β-amyrin) and a 2-methoxy-6-undecyl-1,4-benzoquinone, shown to exhibit anti-inflammatory and antihyperalgesic activities [28].

Preceding phytochemical studies of *Ardisia crispa* roots have reported long chain alkyl-1,4-benzoquinones as major constituents that are potential for phytopharmaceuticals [26, 28, 29]. This plant possesses various pharmacological activities, including antipyretic, antihyperalgesic, anti-inflammatory and antitumor properties [26, 28, 30, 31]. Recently, hexane fraction of the plant roots (ACRH) has been evidenced with potent anti-angiogenic effect in both in vitro and in vivo models of angiogenesis [32, 33]. In the rat model of adjuvant-induced arthritis, ACRH was shown to inhibit pro-inflammatory cytokines, such as TNF-α and IL-1β, partially mediated via their antioxidant properties [34]. However, data on the anti-arthritic efficacy of the plant roots in in vitro studies is yet to be examined. Whilst the previous in vivo studies reporting the anti-arthritic potential of the plant roots (ACRH) mediated in part via its antioxidant and inhibition of pro-inflammatory biomarkers (TNF-α and IL-1β), the present study, targeted on the angiogenesis inhibition, as the early event underlying RA. Moreover, it is ideal to examine the in vitro anti-arthritic effect of *Ardisia crispa* roots in modulating the activities of fibroblast-like synoviocytes (FLS), as the effector cells of RA, before more comprehensive in vivo studies mimicking angiogenic environment took place.

Thus, in this study, we aimed to investigate the anti-arthritic potential of *Ardisia crispa* roots hexane fraction and its benzoquinone constituent mediated by their targeted inhibition on angiogenic human umbilical vein endothelial cells (HUVECs) and human fibroblast-like synoviocytes for rheumatoid arthritis (HFLS-RA) cellular functions in vitro. Considering the significance of angiogenesis inhibition in developing a new anti-arthritic agent, therefore it is warranted for the plant to be screened for its potential via targeted inhibition on angiogenesis.

## Methods

### Chemical and reagents

Human umbilical vein endothelial cells (HUVECs) and endothelial cell medium (ECM) were purchased from ScienCell, USA; Human fibroblast-like synoviocytes for rheumatoid arthritis (HFLS-RA) and synoviocytes growth medium (SGM) were supplied by Cell Applications INC (USA); VEGF_165_ and IL-1β were purchased from Sigma-Aldrich, USA; Suramin hexasodium salt and dimethyl sulfoxide (DMSO) were obtained from Abcam, UK; 3-(4, 5-dimethylthiazol-2-yl)-2, 5-diphenyltetrazoliumbromide (MTT) reagent and phosphate buffered saline (PBS) were purchased from Tocris Bioscience, UK; Matrigel™ (10 mg/mL) and Transwell inserts were obtained from Corning, USA; Ethanol, *n*-hexane, methanol, ethyl acetate, Silica gel 60 (0.063–0.200 mm), and thin-layer chromatography (TLC Silica gel 60 F254) were supplied from Merck, Germany; Whatman filter paper (No. 1) was supplied by Whatman Ltd., England. Hematoxylin and eosin (H & E) stain was supplied from Cellpath, UK; Fluorescein isothiocyanate (FITC) Annexin V Apoptosis Detection Kit I, Acutase™ cell detachment solution, propidium iodide, and calcein AM were purchased from BD Pharmingen™, USA.

### Preparation of bioactive fractions and compound from *Ardisia crispa* roots

*Ardisia crispa* roots were collected from Machang, Kelantan, Malaysia in April 2010 and were authenticated by the botanist at the Herbarium of Universiti Kebangsaan Malaysia, where the voucher specimen was deposited (Reference number of 20841). Dried plant roots (500 g) were finely powdered using a mechanical pulverizer model RT-08 (Rong Tsong Precision Technology Co., Taiwan) and soaked in 70% aqueous ethanol (3 × 2000 mL, 72 h each). The extract was then filtered through Whatman No.1 filter paper and concentrated using a rotary evaporator (Heidolph, Germany) at 40 °C under reduced pressure to yield intermediate *Ardisia crispa* roots aqueous ethanolic extract (ACRE). ACRE was further immersed in an absolute *n*-hexane (3 × 1000 mL, 72 h each), filtered and concentrated under the similar processes to yield *Ardisia crispa* roots hexane extract (ACRH). ACRH was then fractionated into quinone-rich fraction (QRF), and was further isolated to yield benzoquinone compound (BQ) via silica gel column chromatography, adopted via the previous protocol [35]. Briefly, the stationary phase was prepared using amounts of *n*-hexane soaked in silica gel 60 (0.063–0.200 mm) packed into a column (65 cm × 2 cm) and left overnight for the column stability. Approximately 5 g of ACRH (in ratio 1 g of ACRH per 80 g of silica gel) was loaded on top of the packed column. ACRH was then eluted with 500 mL of each mobile phase prepared from gradient mixture of *n*-hexane: ethyl acetate, at the increasing polarity (9:1, 8:2, 7:3, 6:4, and 5:5, respectively). Eluents were collected at a constant rate into numbered vials and air-dried. Further, thin-layer chromatography (TLC) (TLC Silica gel 60 F254) was performed to spot the presence of BQ (compound of interest) in each eluent by calculating their retention factor (Rf) value, as previously described by Hamsin et al. [32]. The estimated Rf value was compared with the Rf value of the reference compound, a 2-methoxy-6-undecyl-1,4-benzoquinone (labeled as BQ, Rf = 0.76), isolated previously by Roslida [36]. Finally, desired fractions containing benzoquinone (BQ) were pooled and re-chromatographed to yield QRF and BQ, respectively.

### GC-MS analyses of ACRH, QRF, and BQ

All samples were then analyzed by gas chromatography-mass spectrometry (GC-MS) for the presence of a major benzoquinone (2-methoxy-6-undecyl-1,4-benzoquinone) and its characterization, to confirm its chemical structure. Composition of benzoquinone in ACRH, QRF, and BQ was analyzed using GC-MS according to method described by Yeong et al. [31] with slight modifications. A 2-methoxy-6-undercyl-1,4-benzoquinone previously isolated by Roslida [36] was included as a reference standard. ACRH and QRF were respectively run on Shimadzu GC-MS QP2010 Ultra system using a capillary column BPX5 (30.0 m × 0.25 mm × 0.25 μm). One microliter (1 μL) of each sample was injected in split mode at an injector temperature of 250 °C. Helium at a constant pressure 37.1 kPa was used as the carrier gas. The injection and the interphase temperature were set at 250 °C. Whilst, the oven temperature was programmed from 50 °C to 300 °C at 3 °C/min, and held for 10 min. The mass spectrometer was an electron impact (EI) ionization mode (70.0 eV). The ion source temperature was set at 200 °C. The benzoquinone was characterized by GC-MS according to the protocol previously described by Yeong et al. [36] and the spectrum was confirmed via comparison with the data obtained [36].

### Cell culture

Human umbilical vein endothelial cells (HUVECs) were cultured in complete endothelial cell medium (ECM) containing 5% fetal bovine serum (FBS), 1% endothelial cell growth supplement (ECGS), and 1% penicillin/streptomycin (P/S). Cells were maintained at 37 °C in a humidified 5% carbon dioxide (CO_2_) incubator. HUVECs above 80% of confluency at passages 3 to 6 were used in this study [37, 38].

Human fibroblast-like synoviocytes for rheumatoid arthritis (HFLS-RA) were cultured in sterile complete synoviocytes growth medium (SGM) supplemented with 100 U/mL 1 penicillin, 80 U/mL 1 streptomycin, 2 mM Gln-glutamine, and 10% FBS. HFLS-RA were maintained at 37 °C in a humidified 5% CO_2_ incubator. HFLS-RA above 80% of confluency at passages 4 to 10 were used in this study [37, 38].

### Cell viability assay

Cell viability of vascular endothelial growth factor (VEGF)-induced HUVECs and interleukin − 1 beta (IL-1β)-induced HFLS-RA post-treated with ACRH, QRF, and BQ were determined using 3-(4,5-dimethylthiazol-2-yl)-2,5-diphenyl tetrazolium bromide (MTT) assay as described by Mossman [39], with some modifications. Briefly, HUVECs (1.5 × 10^5^ cell/mL in complete ECM) and HFLS-RA (5 × 10^4^ cell/mL in complete SGM) were cultured into 96-well plates for overnight attachment. Both cultures were then exposed with various concentrations of the test samples; i.e., ACRH and QRF (1.56, 3.13, 6.25, 12.5, 25, and 50 μg/mL of each sample) and BQ (0.16, 0.31, 0.63, 1.25, 2.5, and 5 μg/mL), respectively, for 24 h in an incubator (37 °C, in 5% CO_2_) along with their respective controls; i.e., negative (untreated) and vehicle (0.1% DMSO). Concurrent to the treatments, both HUVECs and HFLS-RA were induced with VEGF_165_ (20 ng/mL) and IL-1β (10 ng/mL), respectively, to enhance their biological activities [37, 38]. After 24 h of treatment, both cells’ viabilities were determined using MTT-based colorimetric assay according to the kit protocol. Absorbance (optical density) was measured at 570 nm using microplate reader (Tecan M200, USA). The results were expressed as the relative cell viability percentage presented by a reduction in the absorbance after the treatments to the untreated controls by the following equation [40]:

$$ \%\mathrm{of}\ \mathrm{cell}\ \mathrm{viability}=\frac{\mathrm{Absorbance}\ \mathrm{of}\ \mathrm{treated}\ \mathrm{cell}\mathrm{s}\ }{\mathrm{Absorbance}\ \mathrm{of}\ \mathrm{untreated}\ \mathrm{cell}\mathrm{s}} \times 100 $$

Concentration-response curves of the percentage of untreated control versus logarithm of concentration of treatments used were plotted. The number of surviving cells after treatment of ACRH, QRF, and BQ at each concentration was expressed in percentage mean ± SEM, respectively. Graphs of various concentrations against cell viability were plotted from the calculation obtained towards HUVECs and HFLS-RA, respectively. From the curves illustrated, IC_50_ for ACRH, QRF, and BQ against HUVECs and HFLS-RA were respectively obtained. Therapeutic concentrations (0.05, 0.5, and 5 μg/mL of each sample) that were predetermined based on the calculated IC_50_ values were used for the subsequent assays.

### Tube formation assay

Effects of ACRH, QRF, and BQ on VEGF-induced HUVECs tube formation were respectively assessed according to the previous method by Kong [37] with minor modification. Briefly, 96-well plate was precoated with 40 μL of Matrigel (10 mg/mL) and allowed to solidify for 30 min in the incubator at 37 °C and in 5% CO_2_. Next, HUVECs (1.5 × 10^4^ cell/well) in ECM media consists of FBS (5%) and VEGF_165_ (50 ng/mL) were seeded on the Matrigel. Subsequently, the cells were treated with various concentrations (0.05, 0.5, and 5 μg/mL) of ACRH, QRF, and BQ for 16 h, respectively. Concurrent to the treatments, VEGF_165_ (50 ng/mL) was added to all the test samples including the controls; i.e., negative (untreated), positive (suramin 50 μg/mL), and vehicle (0.1% DMSO), to enhance capillary-like formations on Matrigel. After 16 h of treatment, the cells were washed with phosphate buffered saline (PBS) and stained with calcein AM (8 μg/mL) for 30 min (37 °C, in 5% CO_2_), in the dark. Micrographs of the fluorescent labeled capillary-like structures were photographed using inverted fluorescent microscope (Olympus, Japan) at 40 × magnification. To quantify the results, we calculated the total tube length in four random fields of each sample. The assay was repeated three times in triplicate measurements, and the percentage of total tube length was determined by comparing each treatment against the negative control. Data were expressed as the calculated mean ± SEM of the relative tube length.

### Cell invasion assay

Cell invasion assay was performed using Transwell chambers (8.0 μm in pore size) by following the previous protocol with some modifications [38]. In brief, the chambers were precoated with 30 μL of diluted Matrigel (125 μg/mL in SGM media) and incubated overnight at 37 °C, in 5% CO_2_. Prior to the experiment, the Matrigel coatings were rehydrated with SGM media (0.5 mL) for 30 min. Subsequently, IL-1β (10 ng/mL) induced HFLS-RA (1 × 10^4^ cells/well) were seeded and treated with various concentrations (0.05, 0.5, and 5 μg/mL) of ACRH, QRF, and BQ, respectively on the upper chambers. Meanwhile, the lower chambers were filled with SGM containing FBS (10%) plus the equivalent concentrations of the samples as in the upper chambers. VEGF_165_ (10 ng/mL) was added to the lower chambers as chemoattractant. The negative (untreated), positive (suramin 50 μg/mL), and vehicle (0.1% DMSO) were served as controls. Cell invasion was then allowed to occur for 22 h in the incubator (37 °C, in 5% CO_2_). After 22 h of treatment, the non-invasive cells on the top membrane surface were removed. Whilst, the invaded cells on the reverse side of the membrane were fixed with absolute methanol (10 min) followed by hematoxylin and eosin (H & E) staining for 2 min, washed and later photographed. The invaded cells were counted at five random fields under inverted light microscope (Leica, Germany) at 10 × magnification, and analyzed with the aid of ImageJ software. The percentage of invaded cells was calculated by comparing each treatment against the negative control. Data were expressed as the percentage mean ± SEM of the invaded cells.

### Cell apoptosis assay

Apoptotic effects of ACRH, QRF, and BQ on IL-1β-induced HFLS-RA were assayed using fluorescein isothiocyanate (FITC) Annexin V Apoptosis Detection Kit I, adopted from previous work [41], with some modifications. Briefly, HFLS-RA (1 × 10^6^ cell/mL) were cultured into 6-well plate for overnight attachment. Next, the cells were treated with various concentrations (0.05, 0.5, and 5 μg/mL) of ACRH, QRF, and BQ, respectively for 24 h. Concurrently, IL-1β (10 ng/mL) was also added to all test samples including the controls; i.e., negative (untreated), positive (suramin 50 μg/mL), and vehicle (0.1% DMSO), respectively. After 24 h of treatment, treated cells were harvested using Acutase™ cell detachment solution (BD Biosciences, USA), washed with pre-cold PBS (2 ×), and were then centrifuged at 220 × *g* (5 min at 4 °C). The cell pellets (1 × 10^5^ cell/mL) in 100 μL of binding buffer (1 ×) were then stained with the mixture of FITC Annexin V (5 μL) and propidium iodide (5 μL), for 15 min at room temperature in the dark. Lastly, the cell suspensions were analyzed using BD FACSCanto II flow cytometer (BD Biosciences, USA), which, 10,000 cells per sample were acquired for the analysis. The percentage of cell distribution was calculated by comparing each treatment against the negative control. Data were expressed as the percentage mean ± SEM of the apoptotic cells.

### Statistical analysis

SPSS Statistics version 25.0 for Windows (IBM, USA) was used in this study. Data from in vitro experiments were expressed as mean ± standard error of mean (SEM). Three independent experiment (*n* = 3) of each assay were performed in triplicate measurements. The mean difference of cell viability, relative tube length, percentage of invaded cells, and percentage of apoptotic cells post treatment with ACRH, QRF, and BQ were analyzed with one-way analysis of variance (ANOVA) followed by post-hoc test of Tukey’s Honest Significant Difference (HSD), respectively. The significance value was set at *P* < 0.05.

## Results

### GC-MS fingerprinting of ACRH, QRF and characterization of BQ

Extraction of *Ardisia crispa* roots (500 g) using aqueous 70% ethanol, followed by absolute *n*-hexane, yielded approximately 13.64 g (24.0%, w/w) of ACRH. Further fractionation of ACRH through column chromatography yielded 1.99 g (14.6%, w/w) of QRF. Re-chromatographed of the QRF yielded 0.04 g (1.9%, w/w) of BQ. TLC analyses of ACRH and QRF revealed a compound (yellow spot) with a similar retention factor (Rf = 0.76) with the reference compound, along with few other spots (data not shown). Whilst, BQ was observed with a single spot at the similar Rf (0.76) on the TLC plate (data not shown). These observations were parallel to our previous reports [32, 35]. Basically, there were several major and overlapped peaks presented in both ACRH and QRF’s GC-MS finger printings. The presence of the benzoquinone, BQ was observed and identified at Rt = 64.214 min (peak 11) and at Rt = 64.312 min (peak 2) in ACRH and QRF, respectively. Other major peaks assumed to be derivatives of essential oils and phenolic compounds consist in ACRH were identified as diethyl pthalate (peaks 1) in both ACRH and QRF spectra. Hexadecanoic acid (peak 5) and ethyl oleate (peak 8) were only presented in ACRH (Suppl.1A). Whilst, QRF (Suppl. 1B) was shown to consist 5 major peaks, including benzoquinone (peak 2), diethyl phthalate (peak 1), sebacic acid (Peak 5), 2′-dodecyl-5′-allyl-2,5-dimethylpyrrolidine-N-o (peak 6) and two other unidentified compounds at peak 3 and 7, respectively. BQ was further characterized via GC-MS and compared with the data of reference compound [36]. The data of BQ was found to be in agreement with the previous data [36]. The GC-MS spectrum of BQ (Suppl. 2) shared the similarities of the base peak at *m/z* 154 (100) and the ion peak at *m/z* 292 [M^+^], which corresponded to the molecular formula of C_18_H_28_O_3_.

### ACRH, QRF and BQ inhibited VEGF-induced HUVECs and IL-1β-induced HFLS-RA proliferation, respectively

**Fig. 1:**
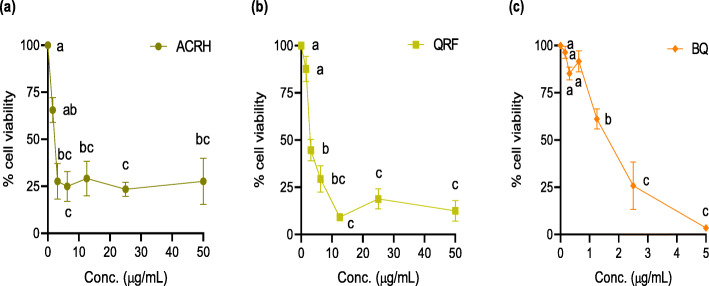
Concentration-response graphs showing the antiproliferative effect of **a** ACRH, **b** QRF, and **c** BQ towards VEGF-induced human umbilical vein endothelial cells (HUVECs) after 24 h of incubation. Data were expressed as percentage mean ± SEM (*n* = 3) of viable cells normalized to 100% cell viability in the negative control and were analyzed using one-way ANOVA followed by Tukey HSD post-hoc test. Significant differences (*P* < 0.05) are indicated by different letters between different concentrations

**Fig. 2:**
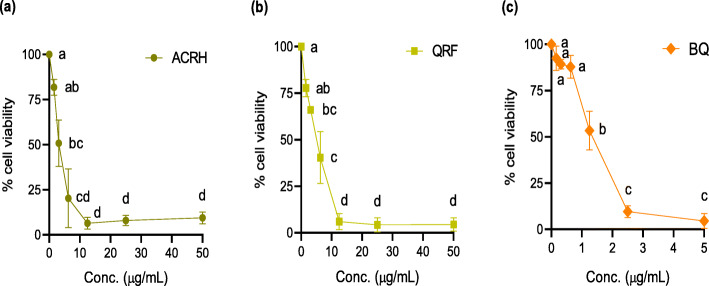
Concentration-response graphs showing the antiproliferative effect of **a** ACRH, **b** QRF, and **c** BQ, respectively towards IL-1β-induced human fibroblast-like synoviocytes for rheumatoid arthritis (HFLS-RA) after 24 h of incubation. Data were expressed as percentage mean ± SEM (n = 3) of viable cells normalized to 100% cell viability in the negative control and were analyzed using one-way ANOVA followed by Tukey HSD post-hoc test. Significant differences (*P* < 0.05) are indicated by different letters between different concentrations

In this study, we determined the antiproliferative effects of ACRH, QRF and BQ on respective VEGF-induced HUVECs and IL-1β-induced HFLS-RA proliferation after 24 h exposure at the range of concentrations used i.e., 1.56–50 μg/mL for ACRH and QRF, respectively and 0.16–5 μg/mL for BQ. As shown in Figs. 1 and 2, treatment with ACRH, QRF, and BQ, respectively, significantly inhibited proliferation of both cells (*P* < 0.05), in a concentration-independent manner. Samples elicited their maximal effects at concentrations close to 10 μg/mL (ACRH and QRF) and 2.5 μg/mL (BQ) in both cells, respectively. After reaching their maximal growth inhibition point, further increment of their concentrations did not seem to cause any greater inhibition towards the cells’ growth (*P* > 0.05). In addition, inhibitory concentration (IC_50_) values of all samples were observed to be below 5 μg/mL (Table 1) in HUVECs and HFLS-RA, respectively, indicating their therapeutic concentration is rather narrow. Based on these MTT results, the concentrations of ACRH, QRF, and BQ used for the subsequent assays were selected at the therapeutic concentrations (0.05 and 0.5 μg/mL) below IC_50_ and a sub-toxic concentration (5 μg/mL) above the IC_50_, respectively. In particular, the concentration above IC_50_ (5 μg/mL) was applied to examine the inhibitory effect of test samples at the sub-toxic concentration on the pathological cells in HFLS-RA. Considering the present MTT data and our previous reports [33, 42], it seems that the sub-toxic concentration of the ACRH and BQ should be within the range of concentrations up to 10 μg/mL.

**Table 1:** The IC_50_ value of ACRH, QRF, and BQ on VEGF-induced HUVECs and IL-1β-induced HFLS-RA

	IC_**50**_ (μg/mL)	
Samples	HUVECs	HFLS-RA
**ACRH**	1.09 ± 0.18^a^	3.60 ± 1.38^a^
**QRF**	3.85 ± 0.26^b^	4.47 ± 0.34^a^
**BQ**	1.34 ± 0.16^a^	1.09 ± 0.09^a^

Values (expressed as mean ± SEM of *n* = 3) with different superscript letters within the same column are statistically different, analyzed by one-way ANOVA followed by Tukey HSD post-hoc test, (*P* < 0.05)

### ACRH, QRF, and BQ inhibited capillary-like tube formation of VEGF-induced HUVECs

**Fig. 3:**
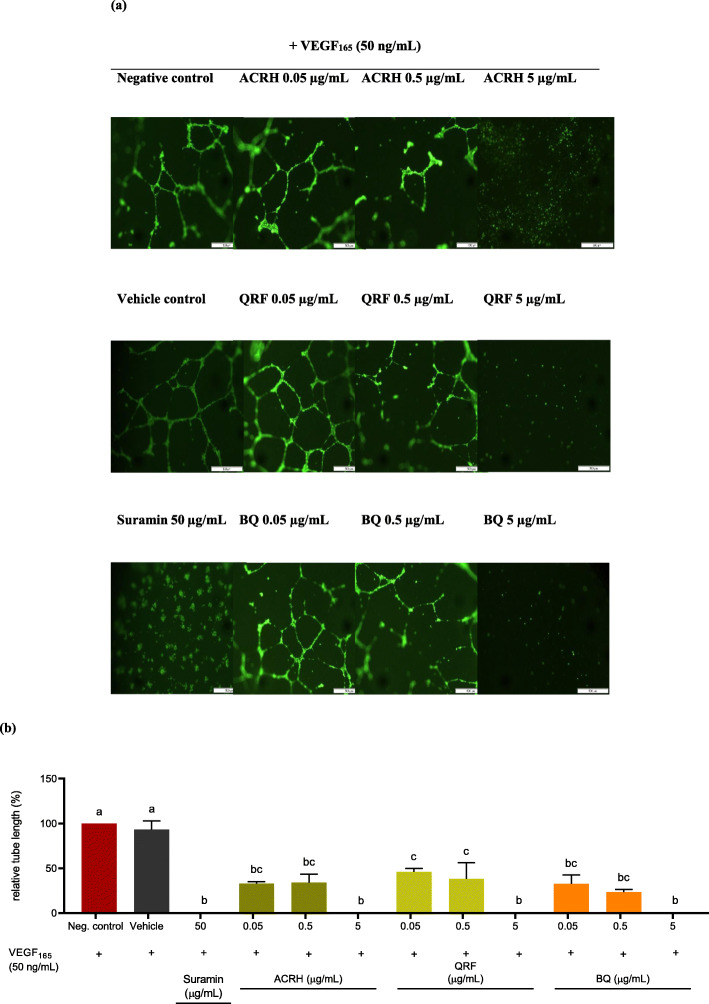
**a** Representative fluorescent images (40 × magnification) showing inhibitory activities of ACRH, QRF, and BQ on HUVECs tube formation after 16 h treatment. **b** Quantitative data of VEGF-induced HUVECs tube formation post treatments with all samples for 16 h. Data were represented as the percentage of tube lengths relative to negative control (normalized to 100% tubular formation) and expressed as mean ± SEM (n = 3). Data were analyzed using one-way ANOVA followed by Tukey HSD post-hoc test. Significant differences (*P* < 0.05) are indicated by different letters between different groups

In this study, we further validated the anti-angiogenic effects of ACRH, QRF, and BQ, respectively via tube formation of HUVECs under influence of excess VEGF (50 ng/mL), assayed on a Matrigel-coated surface. Under the excessive VEGF condition, HUVECs in negative (untreated) and vehicle (0.1% DMSO) controls were observed to form an interconnected network of branching tubules (Fig. 3a), showing 100 ± 0.0% and 93.25 ± 9.61% of relative tube length, respectively (Fig. 3b). Likewise, VEGF-induced HUVECs exposed to ACRH, QRF, and BQ at their respective concentrations (0.05, 0.5, and 5 μg/mL) exhibited significant (*P* < 0.05) reductions in tube length (ACRH: 32.87 ± 2.15%, 33.97 ± 9.33%, and 0.0 ± 0.0%; QRF: 46.06 ± 3.86%, 38.13 ± 18.04%, and 0.0 ± 0.0%; and BQ: 32.71 ± 9.95%, 23.43 ± 2.78%, and 0.0 ± 0.0%), compared to negative control (100 ± 0.0%) (Fig. 3b), after 16 h incubation. However, their inhibitions were equivalent across all samples and were observed in a concentration-independent manner (*P* > 0.05). Also, complete disruption of the tubular networks by these samples at their highest concentration, 5 μg/mL (0.0 ± 0.0%) were comparable to suramin 50 μg/mL (0.0 ± 0.0%) (*P* > 0.05) (Fig. 3b).

### ACRH, QRF, and BQ suppressed cell invasion of IL-1β-induced HFLS-RA

**Fig. 4:**
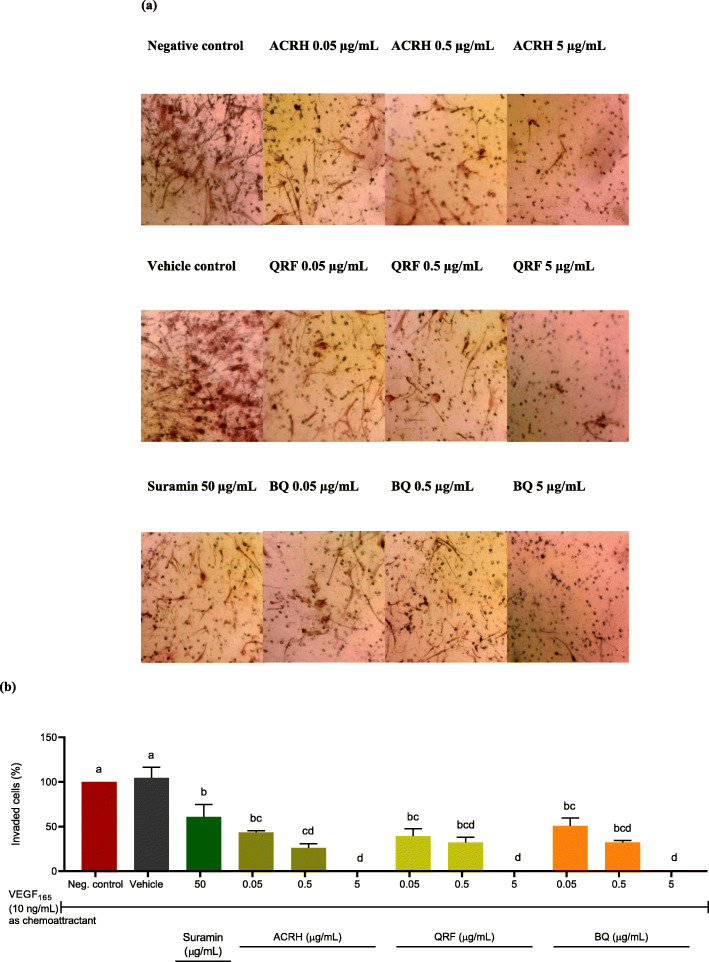
**a** Illustrated micrographs (10 × magnification) showing anti-invasive effects of ACRH, QRF, and BQ on HFLS-RA invasion. **b** Quantitative data of HFLS-RA cell invasion post treatments with all samples for 22 h. Data were represented as the percentage of invaded cells relative to negative control (at 100% invading cells) and expressed as mean ± SEM (n = 3). Data were analyzed using one-way ANOVA followed by Tukey HSD post-hoc test. Significant differences (*P* < 0.05) are indicated by different letters between different groups

This study further examined the anti-invasive effects of ACRH, QRF, and BQ, respectively, on IL-1β-induced HFLS-RA. VEGF_165_ (10 ng/mL) as a chemoattractant was applied on Matrigel-coated inserts (8.0 μm pore size) to mimic the in vivo microenvironment of excessive angiogenesis. As shown in Fig. 4a, HFLS-RA, in response to the VEGF, were highly invasive in both negative (untreated) and vehicle (0.1% DMSO) controls, with 100.0 ± 0.0% and 104.65 ± 11.80% of invaded cells, respectively. As shown in Fig. 4b, no significant difference was observed between both controls, indicating that 0.1% of DMSO used as a vehicle in this experiment was non-reactive by itself (*P* > 0.05). Likewise, IL-1β-induced HFLS-RA treated with ACRH, QRF, and BQ at their respective concentrations (0.05, 0.5, and 5 μg/mL) showed significant (*P* < 0.05) reductions/inhibitions of invaded cells (ACRH: 43.56 ± 1.79%, 26.13 ± 4.64%, and 0.0 ± 0.0%; QRF: 39.44 ± 8.09%, 32.17 ± 5.80%, and 0.0 ± 0.0%; and BQ: 50.84 ± 8.65%, 32.39 ± 1.84%, and 0.0 ± 0.0%), compared to negative control (100 ± 0.0%) (Fig. 4b), after 22 h incubation. However, their suppressions were equivalent across all samples and were observed in a concentration-independent manner (*P* > 0.05). Also, the inhibition of HFLS-RA invasion by these samples at their low (0.05 μg/mL) and mid (0.5 μg/mL) concentration were comparable to suramin 50 μg/mL (60.84 ± 13.81%) (*P* > 0.05) (Fig. 4b). Whilst, the concentration of 5 μg/mL (0.0 ± 0.0%) of all samples resulted in a complete blockade of the HFLS-RA invasion, respectively (Fig. 4b).

### ACRH and BQ significantly promoted apoptosis on IL-1β-induced HFLS-RA

**Fig. 5:**
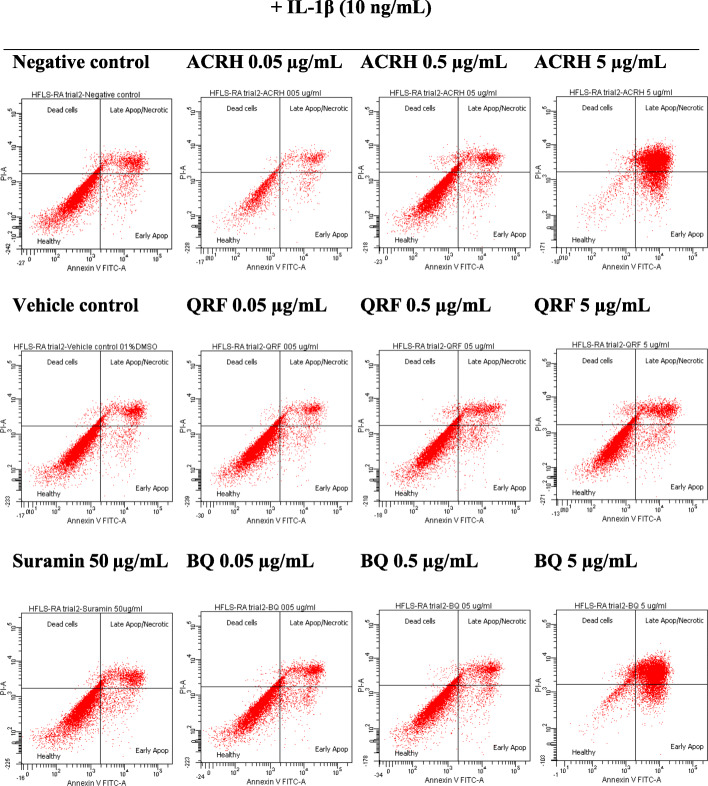
The dot plots showing the cell distribution of IL-1β-induced HFLS-RA as determined by Annexin V-PI staining. The cells were induced with IL-1β (10 ng/mL) and concurrently exposed to various concentrations (0.05, 0.5, and 5 μg/mL) of ACRH, QRF, and BQ respectively for 24 h

In this study, the apoptotic activities of ACRH, QRF, and BQ at their respective concentrations (0.05, 0.5, and 5 μg/mL) on IL-1β-induced HFLS-RA cell apoptosis were assayed in a flow cytometer using FITC Annexin V Apoptosis Detection Kit I. Figure 5 illustrated the cell distribution of IL-1β-HFLS-RA after 24 h treatment with the respective samples. As shown in Tables 2, 24 h treatment with ACRH and BQ at their highest concentration (5 μg/mL) resulted in a significant (*P* < 0.05) increase of cell population in the early (ACRH: 22.7 ± 1.1% and BQ: 8.6 ± 1.7%) and late (ACRH: 50.1 ± 8.6% and BQ: 77.6 ± 6.0%) apoptotic quadrant, respectively, compared to the negative control (early apoptosis: 4.2 ± 0.5% and late apoptosis: 22.8 ± 1.9%). Whilst, their apoptotic activities were insignificant at the low (0.05 μg/mL) and mid (0.5 μg/mL) concentration compared to negative control (*P* > 0.05). In the meantime, treatments with QRF (0.05, 0.5, and 5 μg/mL) and suramin 50 μg/mL for 24 h exhibited a comparable (*P* > 0.05) cell percentage of IL-1β-induced HFLS-RA to the negative control, with less than 20% of cell population were observed within the early and late apoptotic quadrant, respectively (Table 2).

**Table 2:** Percentage of IL-1β-induced HFLS-RA cell population after 24 h of treatment with ACRH, QRF, and BQ respectively

		Cell population (%)			
Samples	Conc. (μg/mL)	Control	Early apoptosis	Late apoptosis	Dead
**Negative control**	**–**	68.9 ± 0.4^a^	4.2 ± 0.5^a^	22.8 ± 1.9^a^	3.6 ± 0.2^ab^
**Vehicle (0.1% DMSO)**	**–**	77.2 ± 0.0^a^	4.6 ± 0.1^a^	15.1 ± 0.3^a^	3.2 ± 0.1^ab^
**Suramin**	**50**	74.1 ± 1.4^a^	6.3 ± 0.8^ab^	17.3 ± 1.8^a^	2.2 ± 0.1^a^
**ACRH**	**0.05**	67.2 ± 2.4^a^	4.0 ± 0.8^a^	23.4 ± 0.1^a^	4.0 ± 0.5^ab^
	**0.5**	76.3 ± 0.9^a^	4.5 ± 0.4^a^	15.5 ± 0.3^a^	4.5 ± 0.2^b^
	**5**	26.8 ± 14.6^b^	22.7 ± 1.1^c^	50.1 ± 8.6^b^	5.0 ± 1.6^b^
**QRF**	**0.05**	73.6 ± 0.1^a^	3.9 ± 1.1^a^	17.3 ± 1.2^a^	3.3 ± 0.6^ab^
	**0.5**	75.7 ± 0.2^a^	5.9 ± 0.1^ab^	14.3 ± 0.0^a^	4.0 ± 0.1^ab^
	**5**	69.0 ± 0.9^a^	5.4 ± 0.1^ab^	19.8 ± 1.6^a^	4.4 ± 0.3^ab^
**BQ**	**0.05**	75.0 ± 1.3^a^	6.3 ± 0.4^ab^	15.2 ± 1.5^a^	3.8 ± 0.1^ab^
	**0.5**	75.0 ± 0.8^a^	5.9 ± 0.8^ab^	15.0 ± 0.5^a^	3.4 ± 0.6^ab^
	**5**	4.4 ± 2.1^c^	8.6 ± 1.7^b^	77.6 ± 6.0^c^	5.1 ± 0.5^b^

Values are expressed in mean ± SEM of triplicate measurement (*n* = 3). Significant differences (*P* < 0.05) are indicated by values with different letters within the same column

## Discussion

Angiogenesis is an important phenomenon in the pathogenesis of some diseases. Excessive angiogenesis in particular has been shown to involve in various diseases such as cancer and rheumatoid arthritis (RA) [7, 9]. Natural plants, in general and their compounds have been reported to act as anti-angiogenic therapies in treating excessive angiogenesis related diseases. Natural compounds such as curcumin and resveratrol have been shown to inhibit angiogenesis in both in vitro and in vivo models by imitating the diseases and suppressing the pro-angiogenic factors such as VEGF and cyclooxygenase (COX)-2 [43, 44]. The strong correlation between angiogenesis and chronic inflammation reflected in both cancer [45] and RA [46] has triggered us to conduct the current study on bioactive extract, fraction, and compound separated and isolated from *Ardisia crispa* roots.

Our group has previously reported various pharmacological activities exerted by ACRH [28, 30, 32–34], QRF [31, 32], and BQ [36, 42, 47] in various in vitro and in vivo models of diseases, some that can be related with excessive angiogenesis, i.e., tumor promotion, inflammation-induced angiogenesis, and arthritis. Nevertheless, the definite involvement of angiogenesis by the root of the plant’s samples in RA were not yet investigated via the particular RA cells, human fibroblast-like synoviocytes for rheumatoid arthritis (HFLS-RA). In addition, we further assessed the samples on human umbilical vein endothelial cells (HUVECs) at lower concentrations, to that of Wen Jun et al. [33] with an additional sample of QRF. This current study first reported on the cytotoxicity of the samples against HFLS-RA and their suppression/inhibition against the cells in angiogenesis in vitro assays.

Angiogenesis, the formation of new blood vessels, is a multistep process involving endothelial cell proliferation, migration, invasion, and differentiation to form tubular structures [48]. Theoretically, inhibition to this coordinated process could be therapeutic at every step of the angiogenic processes. In this study, ACRH, QRF, and BQ, were respectively shown to inhibit proliferation and tube formation of HUVECs under excess VEGF stimulation, mimicking the excessive angiogenesis condition. The present data exhibited lower IC_50_ of ACRH, QRF, and BQ at 1.09 ± 0.18, 3.85 ± 0.26, and 1.34 ± 0.16 μg/mL, respectively (Table 1). The IC_50_ for both ACRH and BQ were in agreement with our previous findings [33]. Nevertheless, the IC_50_ of QRF was found to be higher compared to ACRH and BQ, respectively. Quinones, in general, are cytotoxic agents [49] with selective cytotoxicities against cancer cells [50] than physiological cells. In a different study, Acharya et al. [51] reported a reduced cytotoxicity (IC_50_ = 42.58 ± 1.1 μM) of a benzoquinone derivative, thymoquinone, from *Nigella sativa*, in normal HUVECs, whilst exhibited a selective cytotoxicity to cancer cells, such as A549, MCF7, and Caco2 cells (IC_50_ between 9.67 to 16.4 μM) [51]. Contrary to other studies, our samples (particularly ACRH and BQ) showed reduced IC_50_ in HUVECs (Table 1), which could reflect their narrow therapeutic range in these physiological cells. However, higher IC_50_ of QRF than the former samples could be due to other compounds present in QRF which may antagonize the cytotoxic compound(s).

The HUVECs differentiation into tubular networks under excess VEGF stimulation was also significantly inhibited via ACRH, QRF, and BQ treatments, respectively, at various concentrations as low as 0.05 μg/mL. These data supported the results by Wen Jun et al. [33], which exhibited HUVECs differentiation inhibition by both ACRH and BQ at a slightly higher concentration. Interestingly, QRF was also shown to significantly disrupt the excessive tubular formation of HUVECs (Fig. 3a and b). While the previous work [33, 42] highlighted the anti-angiogenic effect of the samples (ACRH and BQ) in the physiological condition of HUVECs angiogenesis, our present data, particularly on QRF, demonstrated their inhibitory activities against the pathological state of HUVECs angiogenesis stimulated via excessive VEGF, mimicking the RA angiogenesis. These findings thus validated the in vitro anti-angiogenic effect of ACRH, QRF, and BQ from *Ardisia crispa* roots, on HUVECs excessive angiogenesis microenvironment.

Next, we examined the efficacy of ACRH, QRF, and BQ, respectively at the cellular level, by targeting the aggressive features of fibroblast-like synoviocytes (FLS) in HFLS-RA in vitro model. FLS, that line the internal synovium of joints, are aggressive effector cells of RA, which are able to hyperproliferate, highly invasive, and resistant to receptor-mediated apoptosis [52]. In RA, synovial angiogenesis is associated with the chronic inflammation of rheumatoid synovium and this process is hallmarked by high vascularization of the inflamed synovium, allowing infiltration of the inflammatory cells through the vessel wall to the synovial membrane. This resulted in the growth of the aggressive hyperplastic synovial front, called pannus, which invades and destructs the cartilage and bone [53]. Therefore, by controlling this early process in rheumatoid synovium, early prevention in active joints inflammation which eventually leads to destruction in RA patients is necessitated.

RA-FLS via hypoxia-inducible factor (HIF)-1α gene activation can increase VEGF production and the release of other growth factors critical for angiogenesis, including angiopoietin-2, placental growth factors, and fibroblast growth factors [54]. RA-FLS are also a perpetuator of inflammation through the constitutive release of various pro-inflammatory cytokines (e.g., TNF-α, IL-6, IL-17, etc.), and chemokines, such as CXCL1 for inflammatory cell recruitments [55]. As reviewed by Ganesan and Rasool [56], high expression of TNF-α, IL-1β, IL-6, IL-8, IL-15, vascular cell adhesion molecule-1 (VCAM-1), thrombospondin-1 (TSP-1), and stromal cell-derived factor-1 (SDF-1) have been reported in FLS of RA patients. Xing et al. [57] have also reported the upregulation of IL-21 and its receptor (IL-21R) in the synovial tissues and FLS of RA patients, which stimulated the RA-FLS proliferation. Aggressive FLS also increase adhesion molecules (e.g., integrins), enhancing their attachment and invasiveness into the articular cartilage [58]. FLS can produce matrix-degrading enzymes, such as matrix metalloproteinases (MMP)-2 and MMP-9, which promote cartilage matrix degradation [59, 60]. FLS, together with other cytokines release, assist in receptor activator of NF-κB ligand (RANKL) expression of osteoclasts to promote bone erosion [61]. The cytokine profiles and transformed characteristics of FLS in RA make the cells therapeutically targetable. Therefore, many studies have been directed to modulate the aggressive behaviors of FLS in the treatment of RA, ideally using HFLS-RA in vitro model [62–65].

Consistently, we also found antiproliferative effects of ACRH, QRF, and BQ on HFLS-RA proliferation, observed in a concentration-independent inhibition (Fig. 2) and comparable with their antiproliferative effects against physiological HUVECs (Fig. 1). All samples also exhibited low IC_50_ values on HFLS-RA, with ACRH (3.60 ± 1.38 μg/mL), QRF (4.47 ± 0.34 μg/mL), and BQ (1.09 ± 0.09 μg/mL), respectively (Table 1), indicating their considerable increase of cytotoxic activities on these pathological cells. BQ was shown to be highly cytotoxic, evidenced by its lowest IC_50_ amongst all samples. A recent study by Sun et al. [66] reported the low IC_50_ (2.56 ± 0.42 μM) of a quinone derivative, cryoptanshinone from *Salvia miltiorrhiza* Bunge on ROS-induced HFLS-RA. Contrarily, in another study, Umar et al. [67] reported the non-marked effect of thymoquinone in TNF-α-induced HFLS-RA viability, yet the compound was able to inhibit cell adhesion via regulation of apoptosis-regulated signaling kinase 1 (ASK1), indicating its anti-angiogenic effect was not mediated via inflammatory (NF-κB) pathway. Therefore, our current findings may suggest the antiproliferative effects of ACRH, QRF, and BQ against HFLS-RA may mediate via inflammatory pathway, which is in agreement with our previous study [34]. In addition, our group previously reported higher cytotoxicity of ACRH and QRF on Raji cells [35].

Whilst, in other studies using the plant’s leaves, its hydromethanolic and ethyl acetate extracts exhibited moderate cytotoxicity against human breast cancer cells; i.e., MCF-7 and MDA-MB-231 [22]. Moreover, its methanolic extract was shown to exert selective cytotoxicity towards *Mus musculus* mammary carcinoma cell line (4 T1) than the normal fibroblast cell line (NIH3T3) [68]. Remarkably, high cytotoxicity of these samples could be an advantage for suppressing cell survival of pathological cells. These consistent findings thus confirmed the role of *Ardisia crispa* as potent antiproliferative agents with remarkable cytotoxic activities against pathological cells, which may also partly validate for their anti-arthritic properties.

The anti-invasive potential of these samples was further assessed on IL-1β-induced HFLS-RA invasion assay with VEGF acted as a chemoattractant. In this study, HFLS-RA were induced with IL-1β to promote inflammatory responses in RA thus imitating the excessive inflammation in in vivo RA condition. This particular in vitro model may also postulate the therapeutic effect of the tested samples mediated via inflammatory pathway(s) [69]. Post treatment with ACRH, QRF, and BQ respectively suppressed the invasion of IL-1β-induced HFLS-RA in a concentration-independent manner, observed even at the lowest tested concentration (0.05 μg/mL), respectively (Fig. 4). Kang et al. [26] previously reported on the anti-invasive and antimetastatic of 2-methoxy-6-tridecyl-1,4 benzoquinone from the same plant on B16-F10 melanoma cells, in vivo. Whilst, our group also previously reported the anti-invasive of ACRH and BQ on HUVECs, in vitro [33, 42]. These results thus suggested that all samples exhibited their anti-arthritic abilities by inhibiting angiogenesis, manifested by their anti-invasive activities on HFLS-RA. Specifically, BQ may also act as anti-angiogenic compound in treating RA, and potential anti-metastatic compound in cancer therapy, supported by the findings reported by Kang et al. [26]. A study to determine the latter effect in colorectal cancer is now in progress.

Inadequate apoptosis of FLS is generally recognized as one of the characteristics of RA in the disease’s pathological basis. The apoptotic defect of FLS can lead to abnormal synovial hyperplasia, pannus formation, and inflammatory cell infiltration, resulting in cartilage and bone erosion, joint destruction, joint deformity, and eventually joint function loss [70]. Furthermore, there is a close association between angiogenesis activation with apoptosis in RA as reviewed by Middleton et al. [71]. Therefore, anti-angiogenic therapy which is able to effectively promote apoptosis in FLS and inhibit synovial hyperplasia has important clinical significance for RA treatment. We further assessed the potential of ACRH, QRF, and BQ in promoting IL-1β-induced HFLS-RA apoptosis.

Apparently, both ACRH and BQ were found to exhibit significant early and late apoptosis of HFLS-RA, elicited only at their highest concentration (5 μg/mL) (Table 2). Whilst, no significant effect of the QRF observed in this study at all concentrations. Both ACRH and BQ were reported to promote apoptosis in HUVECs [33, 42]. Our current observations were in line with the previous findings [33, 42], which demonstrated the potent proapoptotic effect on physiological (HUVECs) and the current pathological (HFLS-RA) cells. Whilst, no remarkable activity of suramin seen in this assay, signifying its non-apoptotic promoting effect on HFLS-RA. ACRH which contains the benzoquinone along with presence of other phytoconstituents exhibited a potent proapoptotic effect on HFLS-RA. Similarly, BQ also exerted a comparable effect to that of ACRH, thus suggesting that the benzoquinone itself is a potent proapoptotic agent.

Contrarily, QRF did show any significant proapoptotic effect on HFLS-RA. This finding was contraindicated with our previous report on QRF in antitumor promotion, in vivo [72]. Nevertheless, its insignificant proapoptotic effect may also correlate with its highest IC_50_ compared to ACRH and BQ, respectively (Fig. 2, Table 1). We postulated that several constituents present in QRF may antagonize the compound’s (BQ) proapoptotic which may also explain its higher IC_50_ against HFLS-RA. Therefore, additional data should be obtained to generalize the effect of QRF in HFLS-RA apoptosis. While no data was generated pertaining to the cell cycle arrest on QRF, both ACRH and BQ, however, have been previously reported to induce cell cycle arrest of physiological HUVECs at phase G0/G1 [33, 42]. Although we did not examine this effect on this current cell model, however, we postulated that a similar effect might occur in these pathological cells. Nevertheless, a more conclusive explanation to this hypothesis may be attained by examining the cell cycle arrest and morphological analysis of cell death on the pathological FLS.

Developing new therapeutics for angiogenesis-mediated autoimmune inflammatory RA disease needs thorough account on the involvement of multiple signaling pathways; e.g., phosphatidylinositol 3-kinase (PI3K), protein kinase B (AKT), and mammalian target of rapamycin (MTOR) pathways and the cross-talk of related inflammatory cytokines with many other pro-angiogenic factors in this complex disease [73, 74]. Interference to angiogenesis signaling pathways in rheumatoid synovium appears as one of the keys for continuous development of angiogenesis-targeted therapeutics for RA. Accumulating biologics therapies of RA have been directed to target mostly the pro-angiogenic signaling pathways, best known are TNF-α inhibitors (infliximab), anti-IL-1 receptors (Anakinra), and anti-IL-6 receptors (tocilizumab) [75]. Infliximab, a chimeric (mouse-human) monoclonal antibody, is an example of anti-TNF-α that acts by downregulating the pro-inflammatory cytokines, such as IL-1 and IL-6 [76]. This TNF-α inhibitor also reduced serum VEGF in RA patients, as well as exhibiting its clinical efficacy by retarding joint destruction [77]. Anakinra is a recombinant interleukin-1 receptor antagonist (IL-1Ra) that blocks the action of IL-1, a key principal cytokine in inflammation. This anti-IL-1 receptor reduced vessels formation in the pannus of affected joints in RA patients [78]. Another receptor antagonist, tocilizumab, is a potent anti-IL-6 receptor monoclonal antibody that inhibits IL-6 cytokine via its binding to IL-6 receptor. Treatment with tocilizumab has been correlated to reduce angiogenesis in synovial tissues of RA patients [79]. Role of other biologics such as anti-17 antibodies (secukinumab), anti-IL-12/23 antibodies (ustekinumab), anti-CD-20 antibodies (rituximab), anti-B-cell activating factor (belimumab), anti-CD80, and anti-CD86 receptors (abatacept) are also acknowledged for RA therapeutics [75]. To date, the use of these monoclonal antibodies-based therapy in clinical practice, particularly for RA therapies has been growing constantly [80–82].

It is suggested that the mechanistic effect of these test samples, in particular and the plant, *Ardisia crispa* roots, in general may also plausibly modulate RA angiogenesis via similar mechanisms of the aforementioned biologics. Previously, the inhibition of ACRH and BQ on the several pro-angiogenic proteins have been established in HUVECs angiogenesis [33, 42]. Treatment with these samples attenuated expressions of VEGF-C, VEGF-D, Angiopoietin-2, fibroblast growth factor (FGF)-1, Follistatin, hepatocyte growth factor (HGF), and precursor of matrix metalloproteinase 2 (proMMP-2), which were implicated in the cellular process of endothelial cell proliferation, migration, invasion, and differentiation [33, 42], respectively. Therefore, it is postulated that ACRH, QRF, and BQ in this study may also exert anti-angiogenic effects by their suppression on the VEGF-induced phosphorylation of multiple downstream signaling which related to cellular processes of HUVECs angiogenesis.

On the other hand, the role of ACRH in suppressing pro-inflammatory cytokines has been established in in vivo arthritic model. ACRH was previously shown to inhibit TNF-α and IL-1β, as demonstrated in adjuvant-induced arthritis in rat [34]. These pro-inflammatory cytokines play important roles in the synovial inflammation of RA [83]. TNF- α, as a predominant pro-inflammatory cytokine, also plays a role in mediating angiogenesis [84]. TNF-α can induce endothelial cell changes by helping their local release of pro-angiogenic mediators, such as VEGF, basic fibroblast growth factor (bFGF), and platelet-derived growth factor (PDGF) [85], which in turn, encourages the endothelial cell proliferation and migration [86]. TNF-α, together with IL-1β, IL-6, and IL-17 are also known to upregulate angiogenic substances, as seen in pathogenesis of osteoarthritis [85]. Although a conclusive evidence of ACRH, QRF, and BQ acting on in vitro signaling of RA angiogenesis is yet to be defined, however, their inhibition against the in vitro HFLS-RA cellular functions observed in this study is hypothesized via suppression on the IL-1β-mediated activation of signaling pathways associated with the cell proliferation, invasion, and survival of HFLS-RA.

## Conclusion

In summary, our present findings indicated that bioactive fractions and compound of *Ardisia crispa* roots, denoted as ACRH, QRF and BQ significantly inhibited angiogenesis and arthritis in in vitro models, suggesting their anti-arthritic activities are possibly mediated via angiogenesis inhibition. These anti-angiogenic and anti-arthritic effects were reflected by the inhibition on endothelial cell proliferation and tube formation, and the suppression on FLS proliferation, invasion, and apoptotic induction on the FLS, respectively. Clearer explanation on the anti-arthritic activities of these samples on the aforementioned cellular functions might be attained by elucidating detailed mechanism(s) of their action acting on multiple signaling cascades related to angiogenesis in RA.

## Supplementary Information

**Additional file 1.**

**Additional file 2.**

## Data Availability

The data used and analysed in this study are available from the corresponding author on reasonable request.

## Abbreviations

HUVECs: Human umbilical vein endothelial cells
HFLS-RA: Human fibroblast-like synoviocytes for rheumatoid arthritis
VEGF: Vascular endothelial growth factor
IL-1β: Interleukin 1-beta
IC: Inhibitory concentration
ECs: Endothelial cells
FLS: Fibroblast-like synoviocytes
(TNF)-α: Tumor necrosis factor-alpha
RA: Rheumatoid arthritis
ECM: Endothelial cell medium
SGM: Synoviocytes growth medium
DMSO: Dimethyl sulfoxide
MTT: 3-(4,5-dimethylthiazol-2-yl)-2,5-diphenyltetrazolium bromide
PBS: Phosphate buffered saline
H & E stain: Hematoxylin and eosin stain
FITC: Fluorescein isothiocyanate
GC-MS: Gas chromatography-mass spectrometry
FBS: Fetal bovine serum
SEM: Standard error of mean
ANOVA: One-way analysis of variance
Tukey’s HSD: Tukey’s Honest Significant Difference
NSAIDs: Non-steroidal anti-inflammatory drugs
DMARDs: Disease-modifying antirheumatic drugs
COX-2: Cyclooxygenase
FGF-1: Fibroblast growth factor
HGF: Hepatocyte growth factor
proMMP-2: Precursor matrix metalloproteinase 2
bFGF: Basic fibroblast growth factor
COX-2: Cyclooxygenase-2
PI3K: Phosphatidylinositol 3-kinase
AKT: Protein kinase B
mTOR: Mammalian target of rapamycin
HIF-1α: Hypoxia-inducible factor 1-alpha
VCAM-1: Vascular cell adhesion molecule-1
TSP-1: Thrombospondin-1
SDF-1: Stromal cell-derived factor-1
RANKL: Receptor activator of NF-κB ligand
ASK1: Apoptosis signal-regulating kinase 1

## References

[1] Smolen JS, Aletaha D, McInnes IB (2016). Rheumatoid arthritis. Lancet.

[2] Singh JA, Saag KG, Bridges SL, Akl EA, Bannuru RR, Sullivan MC (2016). 2015 American College of Rheumatology Guideline for the treatment of rheumatoid arthritis. Arthritis Care Res.

[3] Henrique da Mota LM, Afonso Cruz B, Viegas Brenol C, Alves Pereira I, Rezende-Fronza LS, Barros Bertolo M (2013). Guidelines for the drug treatment of rheumatoid arthritis. Rev Bras Reumatol.

[4] Xiang G, Jinyu J, Zhitao F, Xiaoqiang H, Yanan L, Zhigang M. A network pharmacology approach to explore the potential targets underlying the effect of sinomenine on rheumatoid arthritis. Int Immunopharmacol. 2020;80(106201). 10.1016/j.intimp.2020.106201. 10.1016/j.intimp.2020.106201 31972421

[5] Tarp S, Furst DE, Boers M, Luta G, Bliddal H, Tarp U (2017). Risk of serious adverse effects of biological and targeted drugs in patients with rheumatoid arthritis: a systematic review meta-analysis. Rheumatology (Oxford).

[6] Szekanecz Z, Koch AE (2009). Angiogenesis and its targeting in rheumatoid arthritis. Vasc Pharmacol.

[7] Carmeliet P (2005). Angiogenesis in life, disease and medicine. Nature..

[8] Volin MV, Koch AE (2011). Interleukin-18: a mediator of inflammation and angiogenesis in rheumatoid arthritis. J Interf Cytokine Res.

[9] Leblond A, Allanore Y, Avouac J (2017). Targeting synovial neoangiogenesis in rheumatoid arthritis. Autoimmun Rev.

[10] Liu S, Li Y, Xia L, Shen H, Lu J (2019). IL-35 prevent bone loss through promotion of bone formation and angiogenesis in rheumatoid arthritis. Clin Exp Rheumatol.

[11] Magro R (2019). Biological therapies and their clinical impact in the treatment of systemic lupus erythematosus. Therapeutic Adv Musculoskeletal Dis.

[12] Šenolt L, Vencovský J, Pavelka K, Ospelt C, Gay S (2009). Prospective new biological therapies for rheumatoid arthritis. Autoimmun Rev.

[13] Baumgart DC, Misery L, Naeyaert S, Taylor PC. Biological therapies in immune-mediated inflammatory diseases: Can biosimilars reduce access inequities? Frontiers in Pharmacology. 2019;10(279). 10.3389/fphar.2019.00279. 10.3389/fphar.2019.00279 PMC6447826 30983996

[14] Moudgil KD, Berman BM (2014). Traditional Chinese medicine: potential for clinical treatment of rheumatoid arthritis. Expert Rev Clin Immunol.

[15] Tabana YM, Al-Suede FSR, Ahamed MBK (2016). Cat’s whiskers (*Orthosiphon stamineus*) tea modulates arthritis pathogenesis via the angiogenesis and inflammatory cascade. BMC Complement Altern Med.

[16] Song X, Zhang Y, Dai E, Wang L, Du H (2020). Prediction of triptolide targets in rheumatoid arthritis using network pharmacology and molecular docking. Int Immunopharmacol.

[17] Nimesh S (2018). Herbal drug is better than allopathic drug in the treatment of rheumatoid arthritis. Int J Pharm.

[18] Avendaño C, Menéndez JC. Drug targeting in anticancer chemotherapy. Med Chem Anticancer Drugs. 2015:595–653. 10.1016/b978-0-444-62649-3.00013-2.

[19] Velderrain-Rodríguez GR, Palafox-Carlos H, Wall-Medrano A, Ayala-Zavala JF, Chen CYO, Robles-Sánchez M, Astiazaran-García H, Alvarez-Parrilla E, González-Aguilar GA (2014). Phenolic compounds: their journey after intake. Food Funct.

[20] Lin D, Xiao M, Zhao J, Li Z, Xing B, Li X, Kong M, Li L, Zhang Q, Liu Y, Chen H, Qin W, Wu H, Chen S (2016). An overview of plant phenolic compounds and their importance in human nutrition and management of type 2 diabetes. Molecules..

[21] De Mejía EG, Ramírez-Mares MV (2011). Ardisia: health-promoting properties and toxicity of phytochemicals and extracts. Toxicol Mech Methods.

[22] Nordin ML, Abdul Kadir A, Zakaria ZA, Abdullah R, Abdullah MNH (2018). *In vitro* investigation of cytotoxic and antioxidative activities of *Ardisia crispa* against breast cancer cell lines, MCF-7 and MDA-MB-231. BMC Complement Altern Med.

[23] Perry LM, Metzger J (1980). Medicinal Plants of East and Southeast Asia: Attributed Properties and Uses.

[24] Chaweewan J, Baumann H, Kenne L, Samuelsson G (1987). Ardisiacrispin a and B, two utero-contracting saponins from *Ardisia crispa*. Planta Med.

[25] Muhammad Z, Mustafa AM (1994). Traditional Malay medicinal plants.

[26] Kang YH, Kim WH, Park MK, Han BH (2001). Antimetastatic and antitumor effects of benzoquinonoid AC7-1 from Ardisia crispa. Int J Cancer.

[27] Huang W (2007). Studies on the antitumor active constituents isolated from the root of *Ardisia crispa*.

[28] Lau MF, Hamid RA, Sabrina S, Nhareet SM (2009). Anti-inflammatory and anti-pyretic effects of hexane fraction of *Ardisia crispa* Thunb.D.C. Pharmacologyonline.

[29] Kobayashi H, de Mejía E (2005). The genus *Ardisia*: a novel source of health-promoting compounds and phytopharmaceuticals. J Ethnopharmacol.

[30] Roslida AH, Kim KH (2008). Anti-inflammatory and anti-hyperalgesic effects of *Ardisia crispa* Thunb D.C. Pharmacogn Mag.

[31] Yeong LT, Hamid RA, Yazan LS, Khaza’ai H (2013). Isolation of a Quinone-rich fraction from *Ardisia crispa* roots and its attenuating effects on murine skin tumorigenesis. Asian Pacific J Cancer Prev.

[32] Hamsin DEZA, Hamid RA, Yazan LS, Taib CNM, Ting YL (2013). The hexane fraction of *Ardisia crispa* Thunb. A DC roots inhibits inflammation-induced angiogenesis. BMC Complement Altern Med.

[33] Wen Jun L, Foong CP, Hamid RA (2019). *Ardisia crispa* root hexane fraction suppressed angiogenesis in human umbilical vein endothelial cells (HUVECs) and *in vivo* zebrafish embryo model. Biomed Pharmacother.

[34] Hamid RA, Fong LM, Ting YL. Anti-arthritic and gastroprotective activities of *Ardisia crispa* root partially mediated via its antioxidant effect. J Complement Integr Med. 2017;15(1). 10.1515/jcim-2017-0012. 10.1515/jcim-2017-0012 28915115

[35] Yeong LT, Hamid RA, Yazan LS, Khaza’ai H, Awang Hamsin DEZ (2014). Synergistic action of compounds isolated from the hexane extract of *Ardisia crispa* root against tumour-promoting effect *in vitro*. Nat Prod Res.

[36] Roslida AH (2004). Anti-inflammatory and analgesic effects of AC-2 isolated from *Ardisia crispa* are mediated via COX-2 inhibition.

[37] Kong X, Zhang Y, Liu C, Guo W, Li X, Su X, Wan H, Sun Y, Lin N (2013). Anti-angiogenic effect of Triptolide in rheumatoid arthritis by targeting angiogenic cascade. PLoS One.

[38] Deng Q, Bai S, Gao W, Tong L (2015). Pristimerin inhibits angiogenesis in adjuvant-induced arthritic rats by suppressing VEGFR2 signaling pathways. Int Immunopharmacol.

[39] Mosmann T (1983). Rapid colorimetric assay for cellular growth and survival: application to proliferation and cytotoxicity assays. J Immunol Methods.

[40] Burton JD (2005). The MTT assay to evaluate chemosensitivity. Methods Mol Med.

[41] Wang YJ, Jiao T, Fu WY, Zhao S, Yang LL, Xu NL (2019). miR-410-3p regulates proliferation and apoptosis of fibroblast-like synoviocytes by targeting YY1 in rheumatoid arthritis. Biomed Pharmacother.

[42] Lim WJ (2017). Antiangiogenic effect of *Ardisia crispa* root hexane extract mediated via its angiogenic signaling cascades.

[43] Bhupinder K, Reena G, Gupta M (2017). Natural products in treatment of rheumatoid arthritis. Int J Green Pharm.

[44] El-Ghazaly MA, Fadel NA, Abdel-Naby DH, Abd El-Rehim HA, Zaki HF, Kenawy SA (2020). Potential anti-inflammatory action of resveratrol and piperine in adjuvant-induced arthritis: effect on pro-inflammatory cytokines and oxidative stress biomarkers. Egyptian Rheumatol.

[45] Aguilar-Cazares D, Chavez-Dominguez R, Carlos-Reyes A, Lopez-Camarillo C, de la Cruz ON H, Lopez-Gonzalez JS (2019). Contribution of Angiogenesis to Inflammation and Cancer. Front Oncol.

[46] Tas S, Maracle C, Balogh E, Szekanecz Z (2016). Targeting of pro-angiogenic signalling pathways in chronic inflammation. Nat Rev Rheumatol.

[47] Awang Hamsin DEZ, Hamid AR, Yazan SL, Taib CNM, Yeong LT (2014). *Ardisia crispa* roots inhibit cyclooxygenase and suppress angiogenesis. BMC Complement Altern Med.

[48] Saranadasa M, Wang ES (2011). Vascular endothelial growth factor inhibition: conflicting roles in tumor growth. Cytokine..

[49] Gutierrez PL (2000). The metabolism of quinone-containing alkylating agents: free radical production and measurement. Front Biosci.

[50] Saibu M, Sagar S, Green I, Ameer F, Meyer M (2014). Evaluating the cytotoxic effects of novel quinone compounds. Anticancer Res.

[51] Acharya BR, Chatterjee A, Ganguli A, Bhattacharya S, Chakrabarti G (2014). Thymoquinone inhibits microtubule polymerization by tubulin binding and causes mitotic arrest following apoptosis in A549 cells. Biochimie..

[52] Pap T, Dankbar B, Wehmeyer C, Korb-Pap A, Sherwood J (2020). Synovial fibroblasts and articular tissue remodelling: role and mechanisms. Semin Cell Dev Biol.

[53] Clarimundo VS, Farinon M, Pedó RT, Teixeira VON, Nör C, Gulko PS, Xavier RM, de Oliveira PG (2017). Gastrin-releasing peptide and its receptor increase arthritis fibroblast-like synoviocytes invasiveness through activating the PI3K/AKT pathway. Peptides..

[54] Bustamante MF, Garcia-Carbonell R, Whisenant KD, Guma M (2017). Fibroblast-like synoviocytes metabolism in the pathogenesis of rheumatoid arthritis. Arthritis Res Ther.

[55] Bartok B, Firestein GS (2010). Fibroblast-like synoviocytes: key effector cells in rheumatoid arthritis. Immunol Rev.

[56] Ganesan R, Rasool M (2017). Fibroblast-like synoviocytes-dependent effector molecules as a critical mediator for rheumatoid arthritis: current status and future directions. Int Rev Immunol.

[57] Xing L, Yang Y, Jin L, Sun C, Li Z, Li J (2016). Interleukin-21 induces proliferation and proinflammatory cytokine profile of fibroblast-like synoviocytes of patients with rheumatoid arthritis. Scand J Immunol.

[58] Firestein GS (2005). Immunologic mechanisms in the pathogenesis of rheumatoid arthritis. J Clin Rheumatol.

[59] Scian R, Barrionuevo P, Giambartolomei GH, De Simone EA, Vanzulli SI, Fossati CA (2011). Potential role of fibroblast-like synoviocytes in joint damage induced by *Brucella abortus* infection through production and induction of matrix metalloproteinases. Infect Immun.

[60] Lee A, Choi SJ, Park K, Park JW, Kim K, Choi K, Yoon SY, Youn I (2013). Detection of active matrix metalloproteinase-3 in serum and fibroblast-like synoviocytes of collagen-induced arthritis mice. Bioconjug Chem.

[61] Kima KW, Choa ML, Lee SH, Oha HJ, Kanga CM, Ju JH (2007). Human rheumatoid synovial fibroblasts promote osteoclastogenic activity by activating RANKL via TLR-2 and TLR-4 activation. Immunol Lett.

[62] Yamanishi Y, Firestein GS (2001). Pathogenesis of rheumatoid arthritis: the role of synoviocytes. Rheum Dis Clin N Am.

[63] Jeoung B, Lee KD, Na C (2013). Ganghwaljetongyeum, an anti-arthritic remedy, attenuates synoviocyte proliferation and reduces the production of proinflammatory mediators in macrophages: the therapeutic effect of GHJTY on rheumatoid arthritis. BMC Complement Altern Med.

[64] Yao Y, Jiang C-S, Sun N, Li W-Q, Niu Y, Han H-Q, Miao ZH, Zhao XX, Zhao J, Li J (2017). Tamaractam, a new bioactive lactam from *Tamarix ramosissima*, induces apoptosis in rheumatoid arthritis fibroblast-like synoviocytes. Molecules..

[65] Li G, Liu D, Guo S, Sunagawa M, Hisamitsu T, Liu Y (2014). Anti-invasive effects of *Celastrus Orbiculatus* extract on interleukin-1 beta and tumour necrosis factor-alpha combination-stimulated fibroblast-like synoviocytes. BMC Complement Altern Med.

[66] Sun HN, Luo YH, Meng LQ, Piao XJ, Wang Y, Wang JR (2019). Cryptotanshinone induces reactive oxygen species-mediated apoptosis in human rheumatoid arthritis fibroblast-like synoviocytes. Int J Mol Med.

[67] Umar S, Hedaya O, Singh HA, Ahmed S (2015). Thymoquinone inhibits TNF-α-induced inflammation and cell adhesion in rheumatoid arthritis synovial fibroblasts by ASK1 regulation. Toxicol Appl Pharmacol.

[68] Nordin ML, Abdul Kadir A, Zakaria ZA, Othman F, Abdullah R, Abdullah MNH (2017). Cytotoxicity and apoptosis induction of *Ardisia crispa* and its solvent partitions against *Mus musculus* mammary carcinoma cell line (4T1). Evid Based Complement Alternat Med.

[69] Liu FF, Chen CH, Chu SJ, Chen JH, Lai JH, Sytwu HK (2007). Interleukin (IL)-23 p19 expression induced by IL-1b in human fibroblast-like synoviocytes with rheumatoid arthritis via active nuclear factor-κB and AP-1 dependent pathway. Rheumatology..

[70] Zhang Q, Liu J, Zhang M, Wei S, Gao Y, Peng W (2019). Apoptosis induction of fibroblast-like synoviocytes is an important molecular-mechanism for herbal medicine along with its active components in treating rheumatoid arthritis. Biomolecules..

[71] Middleton J, Americh L, Gayon R, Julien D, Aguilar L, Amalric F, Girard JP (2004). Endothelial cell phenotypes in the rheumatoid synovium: activated, angiogenic, apoptotic and leaky. Arthritis Res Ther.

[72] Yeong LT, Hamid RA, Yazan LS, Huzwah Khaza’ai H, Mohtarrudin M (2015). Low dose triterpene-quinone fraction from *Ardisia crispa* root precludes chemical-induced mouse skin tumor promotion. BMC Complement Altern Med.

[73] Malemud CJ (2015). The PI3K/Akt/PTEN/mTOR pathway: a fruitful target for inducing cell death in rheumatoid arthritis?. Future Med Chem.

[74] Draoui N, de Zeeuw P, Carmeliet P (2017). Angiogenesis revisited from a metabolic perspective: role and therapeutic implications of endothelial cell metabolism. Open Biol.

[75] Cantatore FP, Maruotti N, Corrado A, Ribatti D (2017). Anti-angiogenic effects of biotechnological therapies in rheumatic diseases. Biologics: Targets and Therapy.

[76] Gibbons LJ, Hyrich KL (2009). Biologic therapy for rheumatoid arthritis. BioDrugs..

[77] Pascual-Salcedo D, Plasencia C, Ramiro S, Nuno L, Bonilla G, Nagore D, Ruiz del Agua A, Martinez A, Aarden L, Martin-Mola E, Balsa A (2011). Influence of immunogenicity on the efficacy of long-term treatment with infliximab in rheumatoid arthritis. Rheumatology..

[78] Dinarello CA (2011). Interleukin-1 in the pathogenesis and treatment of inflammatory diseases. Blood..

[79] Hirohata S, Abe A, Murasawa A, Kanamono T, Tomita T, Yoshikawa H (2016). Differential effects of IL-6 blockade tocilizumab and TNF inhibitors on angiogenesis in synovial tissues from patients with rheumatoid arthritis. Mod Rheumatol.

[80] Curtis JR, Singh JA (2011). Use of biologics in rheumatoid arthritis: current and emerging paradigms of care. Clin Ther.

[81] Balsa A, Tovar Beltrán JV, Cáliz Cáliz R (2015). Patterns of use and dosing of tocilizumab in the treatment of patients with rheumatoid arthritis in routine clinical practice: the ACT-LIFE study. Rheumatol Int.

[82] Wadström H, Frisell T, Askling J (2017). Malignant neoplasms in patients with rheumatoid arthritis treated with tumor necrosis factor inhibitors, tocilizumab, Abatacept, or rituximab in clinical practice. JAMA Intern Med.

[83] Laragione T, Gulko PS (2010). mTOR regulates the invasive properties of synovial fibroblasts in rheumatoid arthritis. Mol Med.

[84] Papetti M, Herman IM (2002). Mechanisms of normal and tumor-derived angiogenesis. Am J Phys Cell Phys.

[85] Wang Y, Xu J, Zhang X, Wang C, Huang Y, Dai K, Zhang X (2017). TNF-α-induced LRG1 promotes angiogenesis and mesenchymal stem cell migration in the subchondral bone during osteoarthritis. Cell Death Dis.

[86] Ushio-Fukai M (2006). Redox signaling in angiogenesis: role of NADPH oxidase. Cardiovasc Res.

