# Supplementary material for: Bioactive fractions and compound of Ardisia crispa roots exhibit anti-arthritic properties mediated via angiogenesis inhibition in vitro
Source: BMC Complement Med Ther. 2021 Jun 25;21:176. doi: 10.1186/s12906-021-03341-y (PMC8235828; doi:10.1186/s12906-021-03341-y)
Supplement: Supplementary file 1 — Additional file 1. [file 12906_2021_3341_MOESM1_ESM.docx]

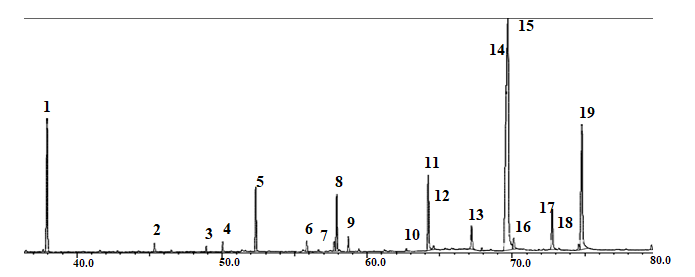


**Supplementary 1A.** Chromatogram of ACRH. (1) Diethyl Phthalate (Rt: 37.95 min), (5) Hexadecanoic acid, ethyl ester (CAS) Ethyl pal (Rt:52.33 min), (8) Ethyl Oleate (Rt: 57.90 min), (11) Benzoquinone (Rt:64.21 min), (13) Phenol, 2-methoxy-5-acetoxymethyl- (Rt:67.20 min) (17) Sebacic acid, di(2,6-dimethoxyphenyl) ester (Rt:72.75 min)


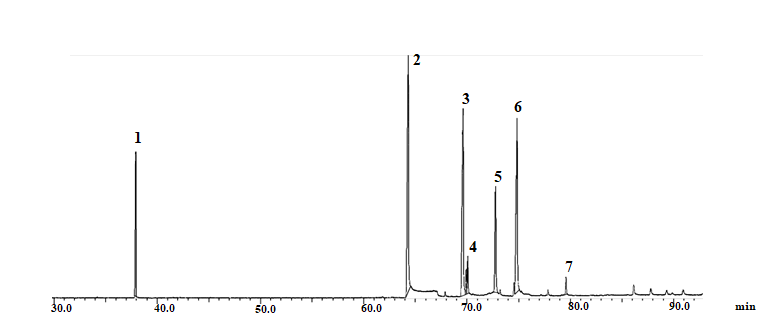


**Supplementary 1B:** Chromatogram of QRF. (1) Diethyl phthalate (Rt:37.93 min), (2) Benzoquinone (Rt:64.31 min), (4) 4A.BETA.,13A.ALPHA.-AZA-4.BETA.-ETHY (Rt:70.06 min), (5) Sebacic acid, di(2,6-dimethoxyphenyl) ester (Rt:72.76 min), (6) 2'-dodecyl-5'-allyl-2,5-dimethylpyrrolidine-N-o. (Rt:74.84 min)
