# Supplementary figures and images for: Bioactive fractions and compound of Ardisia crispa roots exhibit anti-arthritic properties mediated via angiogenesis inhibition in vitro
Source: BMC Complement Med Ther. 2021 Jun 25;21:176. doi: 10.1186/s12906-021-03341-y (PMC8235828; doi:10.1186/s12906-021-03341-y)

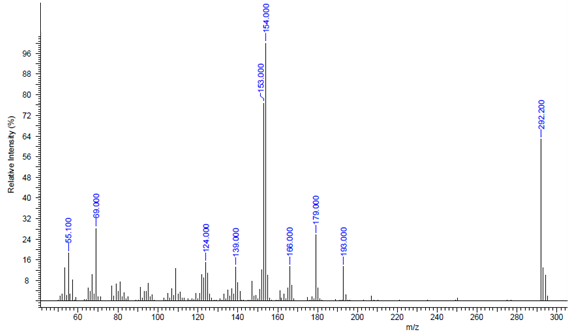


**Supplementary 2**. GC-MS spectrum of 2-methoxy-6-undecyl-1,4-benzoquinone (BQ)

Supplement: Supplementary file 2 — Additional file 2. [file 12906_2021_3341_MOESM2_ESM.docx]
